# Modern contraceptive use and associated factors among married women in Finote Selam town Northwest Ethiopia: a community based cross-sectional study

**DOI:** 10.1186/s40695-018-0044-z

**Published:** 2018-10-19

**Authors:** Alehegn Bishaw Geremew, Abebaw Addis Gelagay

**Affiliations:** Department of Reproductive Health, Institute of Public Health, College of Medicine and Health Science, University of Gondar, 196 Gondar, Ethiopia

**Keywords:** Modern contraceptive, Married women, Ethiopia

## Abstract

**Background:**

A modern contraceptive method is a product or medical procedure that interferes with reproduction following sexual intercourse; however, contraceptive services remain out of reach for many women of reproductive age worldwide, resulting in millions of unwanted pregnancies and unsafe abortions each year. In addition to limiting the number of children, family planning is essential to promoting the well-being and autonomy of women, their families, and their communities. Factors influencing modern contraceptive utilization are multifaceted and challenging, therefore; this study aimed to assess modern contraceptive utilization and associated factors among mid to late reproductive age, married women in Finote Selam town, northwest Ethiopia.

**Methods:**

A community based cross-sectional study was conducted from June 30 to July 15, 2017 among married women aged 30–49. A cluster sampling technique was used to select 1146 eligible participants from three randomly selected kebeles. A face-to-face interviewer administered a structured and pretested questionnaire. Binary logistic regression models, in bivariate and multivariable analyses, were fitted to identify factors associated with the outcome variable. Adjusted odds ratios (AOR) with 95% confidence intervals (CI) was calculated to determine the presence, direction, and strength of associations.

**Results:**

A total of 1134 women aged 30–49 participated in this study representing a response rate of 98.9%.The overall modern contraceptive utilization was 37% (95% CI 35.43–40.21). An injectable contraceptive was the most commonly used method, followed by an implant contraceptive method. Factors independently associated with modern contraceptive use were: educational status -secondary school (AOR = 1.5,95%CI 1.01–2.2) and college and above (AOR = 1.5,95%CI 1.02–2) compared to no education, number of previous pregnancy: nulligravid (AOR = 4.6,95%CI 3.2–5.5),1–2 previous pregnancies (AOR = 3.2,95%CI 2.03–5.44), 3–4 previous pregnancies(AOR = 2.3,95% CI1.4–3.7) compared to > 4 pregnancies and postnatal care utilization (AOR = 1.5,95% CI 1.1–2.1)compared to no postnatal service utilized.

**Conclusion:**

Our findings show that modern contraceptive utilization among women age 30–49 is low in Finote Selam town Northwest Ethiopia. Women’s educational status, low number of previous pregnancies and postnatal care service utilization during the last birth were independently associated with modern contraceptive method used. Providing modern contraceptives targeting grand multiparous women and women having no formal education is important. Improving postnatal care utilization is one potential strategy to enhance modern contraceptive utilization.

## Background

Modern contraceptive methods are products or medical procedures that interfere with reproduction from the act of sexual intercourse [1]. In spite of progress on increasing modern contraceptive service utilization in recent years, contraceptive services remain out of reach for many women of reproductive age worldwide, resulting in millions of unwanted pregnancies and unsafe abortions each year [2]. In addition to limiting the number of children, family planning is essential to promoting the well-being and autonomy of women, their families and their communities [3]. Family planning services are one of the strategies for preventing more than 20% of maternal mortality and 17% of neonatal mortality [4].

In 2015 only 57% of married or in-union women of reproductive age used a modern method of family planning worldwide, with female sterilization (19%) and Intra- Uterine Contraceptive Device (IUCD) (14%) the most commonly used methods [5]. According to the 2016 Ethiopian Demographic Health Survey (EDHS), 35% of currently married women used a modern contraceptive, 23% used an injectable contraceptive and 8% an implant [6]. The factors that influence modern contraceptive utilization are multifaceted and challenging. Several studies provide evidence that modern contraceptive utilization is associated with socio-demographic, socio-cultural and economic factors, including women’s educational status, monthly family income, women’s empowerment, having attended four or more antenatal care visits, and knowledge of family planning [7–10]. Evidence from Nigeria suggests that modern contraception use among grand multiparty women is low and the main reason for non-use is the desire for more children [11]. However, contraceptive use among Human Papiloma virus(HIV) positive grand multiparous women is high [12].The proportion of modern contraceptive use among currently married reproductive age women in the Amhara region reported in the EDHS 2016 was 47% but determinants of use are not available. Moreover information on utilization of modern contraceptive methods among currently married/union women in mid to late reproductive age in the study area is scarce. Women in late reproductive age are more at risk of pregnancy related complications and contraceptive utilization is the best intervention to improve women health.

Therefore our study aimed to assess modern contraceptive utilization and associated factors among married women of middle and late reproductive age in Finote Selam town, northwest Ethiopia.

## Methods

### Study design and setting

A community-based cross-sectional study was conducted from June 30 to July 15, 2017, among married women in Finote Selam town, northwest Ethiopia. Finote Selam town is located in West Gojam Administration Zone of the Amhara Regional State, in northwest Ethiopia. We selected Finote Selam purposely as it is similar to other towns in the Amhara region in socioeconomic status, education, ethnicity and religion. According to the population projections of Ethiopia for all regions at the district level for 2017, the total population of the town was estimated to be 38,399. Out of these,19,923 are male and 18,476 female [13]. The town has five kebeles, the smallest administrative units. Based on information obtained from town administrative the total number of households was 5530.

According to information obtained from the Finote Selam district health office, the district has one primary hospital, one public health centre, four private clinics and five health posts owned by the government. Family planning and maternal health services are provided free of charge in the government health facilities. The sample population for this study was all married women aged 30–49 years living in Finote Selam town.

### Sample size and sampling procedure

The sample size for prevalence of use was determined using a single population proportion formula with the assumptions of 95% confidence interval, 5% margin of error, and assuming 47% used a modern contraceptive in the Amhara region based on the EDHS 2016 [5]. Sample size calculations for assessing factors associated with modern contraceptive utilization [8], assuming a 95% level of confidence, and 80% power resulted in a required sample size of 546, greater than the sample size required for estimating prevalence of use. Assuming 5% non-response rate and adding two design effects, yielded a sample size of 1146 for this study.

Three of the five kebeles were selected by simple random sampling. A cluster sampling technique was used to ascertain study participants. The total sample size was proportionally allocated to the three cluster kebeles based on the number of households within the kebele: 608, 304 and 234 women were allocated to each selected cluster.

### Data collection

A face-to-face interviewer-administered, structured and pretested questionnaire adapted from a previous study was used [9]. The questionnaires were prepared in English and then translated into Amharic, a local language. The questionnaire consisted of items assessing socio-demographic characteristics, reproductive history, maternal health service utilization, and modern contraceptive history. Six female diploma and two BSc degree graduated midwives who could speak the local Amharic language were recruited for data collection, and supervision, respectively. To ensure data quality, 2 days of training on data collection techniques was given to data collectors and supervisors. Interview was conducted at each study participants home or women at shop with in home compound. The data collection and supervision were overseen by researchers to ensure completeness and consistency of data.

### Variable definitions

Modern contraception methods are defined to include female and male sterilization, oral hormonal pills, the intra-uterine device (IUD), male and female condoms, injectables, the implant, vaginal barrier methods and emergency contraception [6].

Antenatal care utilization was defined as having had at least one visit by a skilled provider during the last pregnancy. Postnatal care utilization was defined as having had at least one visit by a health provider during the post-partum period (within 6 weeks after child birth) after the last childbirth.

### Data analysis

The data were checked for completeness, coded manually, and experienced clerks entered data into EPI-info version 7 statistical software. After checking consistency and completeness, data were exported to SPSS version 20 for further analysis. Both bivariate and multivariable logistic regression models were estimated to identify variables associated with modern contraceptive method utilization. All variables with a *p*-value of less than 0.2 in the bivariate analysis were considered for multivariable analysis to control potential confounders.The strength of association was interpreted using adjusted odds ratio with 95% confidence interval.

## Results

### Socio-demographic characteristics of the participants

A total of 1134 of the 1146 eligible women were interviewed for this study giving a response rate of 98.9%. The mean age of the participants was 37.4 years (SD + 5.7 years). Nearly two-thirds (64.9%) of participants were aged 30–39 years. Orthodox Christians accounted for (92.2%) of the study participants. Approximately half (45.1%) of the women had no formal education while 11.5% had attended college or above. Regarding the occupational status of the participants, 47.8% were unemployed and 15.1% were employed in government or private sectors. Nearly 65% of the participants had an average family monthly income of less than $68 US (Table 1).

**Table 1:** Socio-demographic characteristics of study participants in Finote Selam town, Northwest Ethiopia, 2017

Characteristics		Frequency *n* = 1134	Percent
Age	30–39	736	64.9
	40–49	396	35.1
Religion	Orthodox	1046	92.2
	Muslim	80	7.1
	Protestant	8	0.7
Educational status	No formal education	512	45.1
	Primary school	293	25.8
	Secondary school (9–12)	198	17.5
	College/university	131	11.6
Occupational status	Unemployed	542	47.8
	Self employed	421	37.1
	Government/private employed	171	15.1
Family average monthly income^a^	< 68	732	64.6
	68+	402	35.4

^a^ in US dollars

### Reproductive characteristics and use of maternal health services during the last pregnancy

A total of 1013 (89.3%) women had a history of at least one pregnancy and 232 (20.4%) had had five or more pregnancies. Among participants who had a history of pregnancy, 582 (57.4%) had had at least one antenatal visit during their last pregnancy. Antenatal care (ANC) visits were frequently conducted at the hospital. Among women with a history of pregnancy 163 (16.3%) had had at least one abortion. Among women who had experienced an abortion, 75% and 25% were spontaneous and induced abortion respectively. Most participants had a history of at least one childbirth (88.2%) and 19.3% had had five or more children (Table 2) with 38.4% having delivered their last child at home.

**Table 2:** Reproductive characteristics and use of maternal health services during the last pregnancy among married women in Finote Selam town, northwest Ethiopia, 2017

Characteristic		Frequency (*N* = 1134)	Percent
History of pregnancy	No	121	10.7
	Yes	1013	89.3
Number of previous pregnancies	0	121	10.7
	1–2	363	32
	3–4	418	36.9
	5+	232	20.4
Antenatal Care utilization (*N* = 1013)	No	431	42.6
	Yes	582	57.4
Place of Antenatal Care (*N* = 582)	Health center	271	46.6
	Hospital	311	53.4
History of abortion (*N* = 1013)	No	850	83.9
	Yes	163	16.1
How the termination occurred (*N* = 163)	Spontaneous	122	75
	Induced	41	25
History of child birth	No	134	11.8
	Yes	1000	88.2
Parity	0	134	11.8
	1–2	389	34.3
	3–4	392	34.6
	5+	219	19.3
Place of delivery last child *N* = 1000	Home	384	38.4
	Health center	228	22.8
	Hospital	370	37
	Others ^a^	18	1.8
Postnatal Care (*N* = 1000)	No	742	74.2
	Yes	258	25.8
Place of Postnatal Care (*N* = 258)	Health center	145	56.2
	hospital	110	42.6
	Private clinic	3	1.2

^a^ Health post and private clinic

### Modern contraception utilization

**Fig. 1:**
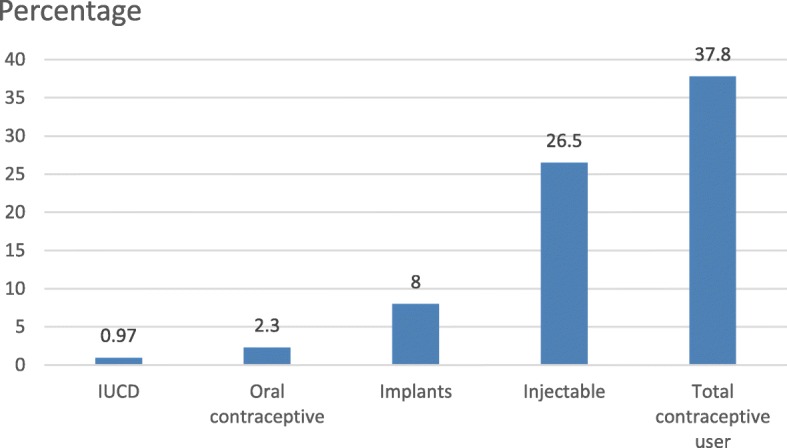
Proportion using each modern contraceptive method among married or in-union women in Finote Selam town, northwest Ethiopia, 2017

Among the participants, 830 (73%) ever used any modern contraceptive method. During data collection, 429 (37.8%) (95% CI 35.4–40.2) were using any modern contraceptive method currently. The most frequently used contraceptive method was an injectable contraceptive (26.5%) followed by implants (8%) with the least used method an intrauterine contraceptive device (IUCD) (0.97%) (Fig. 1).The major (85%) source of contraceptives was a public health facility. Among modern contraceptive users, 70.1% used injectable contraceptives and 21.3% used implant contraceptive methods whereas oral contraceptive and IUCD were used by 6%and 2.6% respectively.

Among women aged 30–39 years, 66% reported using modern contraceptive methods during the survey, whereas only 31.3% of women aged 40–49 were using modern contraceptive methods.

### Determinants of modern contraception use

In the multivariable logistic analysis, higher educational status, lower gravidity, and antenatal care service use were significantly and independently associated with modern contraceptive methods utilization (Table 3).

**Table 3:** Unadjusted and adjusted odds ratios for factors associated with modern contraceptive use among married women in Finote Selam town, northwest Ethiopia, 2017

Factors		Contraceptive utilization		Unadjusted OR 95%CI	Adjusted OR 95%CI
		No	Yes		
Age	30–39	262 (44%)	333 (66%)	1.81 (1.32–2.54)**	1.28 (0.89–1.86)
	40–49	137 (58.7%)	96 (31.3%)	1	1
Educational status	No formal education	176 (57%)	132 (43%)	1	1
	Primary school	118 (48.5%)	125 (51.5%)	1.37 (1.10–1.98)**	1.24 (0.86–1.78)
	Secondary school (9–12)	53 (33%)	107 (57%)	2.69 (1.83–4.01)***	1.53 (1.01–2.24)
	College/university	52 (44%)	65 (56%)	1.67 (1.15–2.55)**	1.51 (1.02–2.03)
Occupation	Unemployed	193 (54.8%)	159 (45.2%)	1	1
	Self employed	60 (41%)	86 (59%)	1.73 (1.13–2.14)**	1.12 (0.65–1.92)
	Government/private employed	146 (44%)	184 (56%)	1.52 (1.82–2.53**	1.31 (0.72–2.36)
History of childbirth	No	35 (30.7%)	79 (69.3%)	1	1
	Yes	364 (51%)	350 (49%)	0.35 (0.22–0.55)	0.24 (0.09–0.23)
Number of previous pregnancy	0	28 (27%)	76 (73%)	7.31 (4.14–13.21)***	4.61 (3.20–5.52)***
	1–2	134 (44%)	170 (56%)	3.40 (2.13–5.40)***	3.22 (2.03–5.44)***
	3–4	148 (49.6%)	150 (50.4%)	2.71 (1.70–4.32)***	2.33 (1.41–3.72)**
	5+	89 (73%)	33 (27%)	1	1
Place of ANC	Health center	101 (44.3%)	12,755.7%)	1.55 (1.13–2.24)**	1.25 (0.92–1.85)
	Hospital	148 (55%)	120 (45%)	1	1
Abortion history	No	328 (46.5%)	376 (53.5%)	1	1
	Yes	71 (57%)	53 (43%)	0.65 (0.44–0.95)**	0.87 (0.72–1.12)
Types of abortion	Spontaneous	56 (65%)	30 (35%)	0.39 (0.17–0.85)**	0.41 (0.18–1.05)
	Induced	16 (42%)	22 (58%)	1	1
Parity	0	35 (30.7%)	79 (69.3%)	5.60 (3.12–7.81)**	2.6 (0.98–6.79)
	1–2	136 (42.6%)	183 (57.4%)	3.34 (2.11–5.31)***	1.99 (0.89–4.17)
	3–4	146 (52%)	134 (48%)	2.28 (1.41–3.60)***	1.7 (0.80–3.34)
	5+	82 (72%)	33 (28%)	1	1
PNC	No	304 (50%)	304 (50%)	1	1
	Yes	95 (36.8%)	163 (63.2%)	1.71 (1.14–2.16)**	1.53 (1.12–2.10)**

*OR* Odds ratio, *CI* Confidence interval, *ANC* Ante-Natal care, *PNC* Post-Natal care, 1: Reference category, *:0.05 ≤ *p* < 0.2, **:0.001 < *p* < 0.05,***:*p* < 0.001

**Fig. 2:**
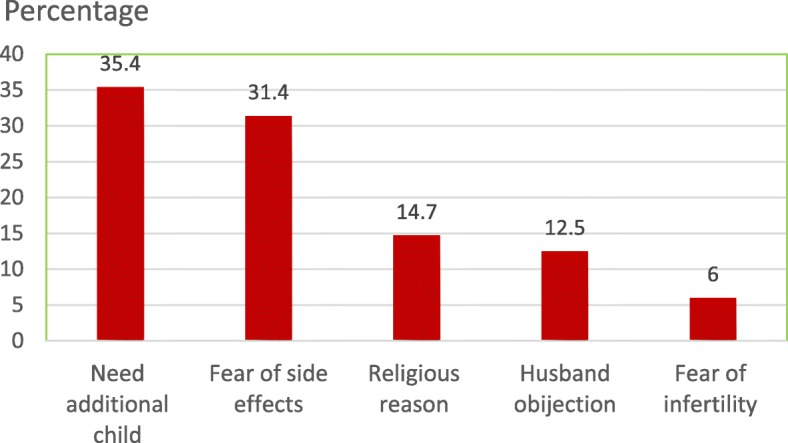
Major reason women were not using a modern contraceptive method among married or in-union women in Finote Selam town, northwest Ethiopia, 2017

Women who had attended secondary school were 1.53 times (95% CI: 1.01–2.24) and who had attended college and above were also 1.51 times (95% CI: 1.02–2.03) more likely to use modern contraceptive methods than women with no formal education. Null gravida participants were 4.61 times (95% CI: 3.20–5.52) more likely to use contraceptive methods than women who had had five or more pregnancies. Whereas those with 1–2 pregnancies were 3.22 times (95%CI: 2.03–5.44) and women with 3–4 pregnancies were 2.33 times (95%CI: 1.41–3.72) more likely to use modern contraceptive methods than women with five or more pregnancies. Women who had postnatal care during the last pregnancy were 1.53 times (95%CI: 1.01–2.10) more likely to use modern contraceptive methods than women who had not (Table 3). Women’s reasons for not using modern contraceptive methods included that they wanted an additional child and fear of side effects of contraceptive methods (Fig. 2).

## Discussion

Family planning services are a cost-effective strategy for preventing more than 20% of maternal mortality and 17% of neonatal mortality by preventing risky and unwanted pregnancy [4]. Use of modern contraceptive methods are especially important to decrease maternal mortality at the two extremes of reproductive age (< 20 years and > 40 years); however the use of modern contraceptive methods among late reproductive age women is low. Therefore the present study assessed modern contraceptive utilization and associated factors among married women in middle and late reproductive age living in Finote Selam town, northwest Ethiopia.

The findings of this study showed that 37.8% of the participants, were currently using modern contraceptive method which is comparable to the 2016 EDHS that reported 35% [6], and the Kersa Demographic and Health Surveillance System of Ethiopia that reported 40% [14]. This utilization is higher than that reported from the Afar region of Ethiopia 20.8% [15], and in rural Mozambique 23% [16]. However, it is lower than that reported from 2016 EDHS Amhara region 47% [6], Kenya 68.2% [17], western Ethiopia 71.9% [7], and urban Kenya 65% [18]. Among the total study participants, 26.5% and 8% were using injectable and implants contraceptive methods respectively. According to the 2016 EDHS the most commonly used methods were also injectable(23%) and implants(8%) [6]. Studies conducted in the other parts of Ethiopia also found that the most commonly used methods among contraceptive user were injectables (78%) and implants (20%) [8, 19], consistent with our findings among contraceptive users.

Previous studies found that women in late reproductive age use modern contraceptive method less than young and middle aged women [12], however age was not independently associated with modern contraceptive use in this study. In our study, women who had secondary school or higher were more likely to use modern contraception methods than those who had no a formal education similar with findings reported in from other studies [8, 19–21]. Our finding suggest that women’s previous pregnancy history was associated with likelihood of modern contraceptive utilization, as nulligravid participants were much more likely to use a modern contraceptive method compared to grand multiparous women. Similar findings were reported in Ethiopian Demographic Health Survey (EDHS 2016) [6].

Antenatal care service utilization and institutional delivery during the last pregnancy were not found to be significantly associated with modern contraceptive utilization in our study. This result is inconsistent with another Ethiopian study, which reported that women who uses antenatal care services and a health facility delivery during the last pregnancy were more likely to use modern contraceptive methods than women who had not used antenatal care [22]. We identified that participants who had utilized postnatal care following their last childbirth were more likely to use any modern contraception methods than women who did not have a postnatal follow-up. This finding is supported by other studies [19, 23].

Reasons that women reported for not using modern contraceptives in our study, including the need an additional child and fear of contraceptive side effects are similar to those reported in north Shoa Ethiopia [9] and Nigeria [11].

Limitations of this study include that the study was carried out in an urban setting and may not be representative of rural women, however, we did include a semi-urban kebele. We have selected Finote Selam purposely, regarding characteristics of the population: socioeconomic status, education, ethnicity and religion it is similar with other towns in the Amhara region of Ethiopia; however, there is a difference with other towns outside Amhara region especially in less developed regions of Ethiopia. Thus our finding is possible to generalize regarding modern contraceptives used among middle and late reproductive age women in the Amhara region in Ethiopia rather than Ethiopia as a whole. Also male involvement was not considered as a determinant in contraceptive utilization.

## Conclusion

This study documented that modern contraceptive utilization among women age 30–49 is low in Finote Selam town Northwest Ethiopia. Increasing women’s educational status, lower number of previous pregnancies and postnatal care service utilization during their last birth were significantly and positively associated with modern contraceptive method use. Providing modern contraceptive services targeting grand multiparous women and women who have no formal education, using community health workers is important. Improving postnatal care utilization is another important strategy to enhance modern contraceptive method uptake.

## Abbreviations

ANC: Ante Natal care
AOR: Adjusted Odds Ratio
CI: Confidence Interval
PNC: Post Natal care
SD: Standard Deviation
SPSS: Statistical Package for Social Sciences
